# Long-term follow-up of blood pressure and glomerular filtration rate in patients with a solitary functioning kidney: a comparison between Wilms tumor survivors and nephrectomy for other reasons

**DOI:** 10.1007/s00467-015-3215-2

**Published:** 2015-10-19

**Authors:** Annelies M. C. Mavinkurve-Groothuis, Frank van de Kracht, Rik Westland, Joanna A. E. van Wijk, Jacqueline J. Loonen, Michiel F. Schreuder

**Affiliations:** Princess Maxima Center for Pediatric Oncology, Utrecht, The Netherlands; Department of Pediatric Hematology and Oncology, Radboudumc Amalia Children’s Hospital, Nijmegen, The Netherlands; Radboudumc Amalia Children’s Hospital Department of Pediatric Nephrology 804, Radboud University Medical Center, PO Box 9101, 6500 HB Nijmegen, The Netherlands; Department of Pediatric Nephrology, VU University Medical Center, Amsterdam, The Netherlands

**Keywords:** Wilms tumor, Solitary functioning kidney, Glomerular hyperfiltration, Blood pressure, Proteinuria

## Abstract

**Background:**

Children with unilateral Wilms tumor (WT) treated with chemotherapy and/or radiotherapy and nephrectomy have excellent survival rates. A solitary functioning kidney (SFK) is associated with progressive renal injury. This study aims to investigate the additional effect of Wilms tumor treatment on renal function compared with children with an SFK for non-oncological reasons.

**Methods:**

A single-center retrospective cohort study on the renal injury markers of 79 survivors of unilateral WT was performed and compared with a matched group of children with an SFK for non-oncological reasons. Mean age at follow-up was 12.4 (SD 5.9) years.

**Results:**

During follow-up, mean estimated glomerular filtration rate (eGFR) and blood pressure z-scores remained stable at an acceptable level. However, in the group of 31 WT patients with a follow-up of 15 years, 23 % showed signs of renal injury. This proportion was smaller than the 54 % in a group of SFK patients based on non-oncological causes (*p* = 0.004).

**Conclusions:**

A significant proportion of WT survivors develop renal injury during follow-up. Our data may be an underestimation of the true frequency of progressive renal injury, due to a lack of information on proteinuria. As with patients with a non-oncological SFK, long-term follow-up is essential to monitor WT survivors.

## Introduction

In recent years, it has become clear that a solitary functioning kidney (SFK) from birth or acquired during childhood leads to progressive renal injury [1, 2]. In the Kidney of MONo-functional Origin (KIMONO) study cohort of over 400 children with an SFK, one in three children showed signs of progressive renal injury (hypertension, proteinuria, and/or drugs prescribed for either of the two indications) during childhood [1, 2]. As described by Brenner et al. [3], this is hypothesized to be based on glomerular hyperfiltration, which results in glomerular hypertension and damage. In addition, a long-term follow-up study of patients with an SFK showed that 20–50 % were dependent on renal replacement therapy at the age of 30 years [4]. These data illustrate that an SFK in childhood may have serious long-term consequences.

One of the reasons for an acquired SFK is a unilateral Wilms tumor (WT). It accounts for 6 % of all childhood malignancies, with the highest incidence rate between 2 and 5 years of age, and a rare occurrence in adolescents or adults [5]. The outcome of patients with WT has significantly improved over the past decades. Five-year overall survival estimates now range from 80 to over 95 %, depending on the stage and histology [6–8]. Chemotherapy and radiotherapy may be harmful to the remaining kidney, but in general the decrease in renal function is believed to be temporary and reversible, and only present during chemotherapeutic treatment [9]. The hyperfiltration hypothesis can also be expected to apply in WT survivors. Green recently reviewed studies on renal dysfunction in WT survivors [9]. Unfortunately, these studies are small with heterogeneous patient populations and with different methods of measuring renal function. In addition, in most SFK follow-up studies, WT patients are excluded, which makes the long-term outcome of having an SFK after tumor nephrectomy together with chemo- and/or radiotherapy compared with SFK after non-oncological causes unknown. We hypothesize that children with an SFK after WT have a decreased renal outcome compared with non-WT post-nephrectomy subjects, resulting in a higher rate of hypertension, proteinuria, and/or reduced glomerular filtration rate (GFR). This study aims to investigate the additional effect of WT treatment on long-term renal function compared with children with an SFK after nephrectomy for non-oncological reasons.

## Materials and methods

### Patients

All survivors of unilateral WT, diagnosed between 1987 and 2011 at the Radboud University Medical Center from whom follow-up data were available, were included in our study. Data were retrospectively collected at four set time-points, i.e., after 2.5, 5, 10, and 15 years of follow-up, and were compared with those of matched children with SFK for non-oncological reasons from the KIMONO cohort [2]. Patients with a renal tumor other than WT were excluded from the study, as were patients with associated syndromes such as WAGR (WT, aniridia, genitourinary malformation, and mental retardation) and Denys–Drash syndrome.

### Data collection

All data were collected from the patient records using a case record form. Tumor and treatment characteristics were collected from each patient. At diagnosis and at every follow-up time point, medical examination, laboratory and/or radiology data were recorded. During follow-up, standard medical examination and renal laboratory testing (serum creatinine, urine albumin–creatinine or protein–creatinine ratio or urine dipstick) were collected when available. Different methods of determining creatinine levels were used. Unfortunately, there were insufficient data on proteinuria and renal length to evaluate as a separate marker. When no data were available, these were considered to be within the normal range to prevent the overestimation of renal injury. The GFR was estimated (eGFR) using the revised Schwartz formula: eGFR (ml*min^−1^*1.73 m^−2^) = *k* × height (m)/serum creatinine [in mg/dL]), with a *κ* value of 41.3 [10]. Blood pressure (BP) was measured using either a manual auscultatory or an automatic oscillometric device. Neither the method nor the device used was specified in the patient file. High BP was defined as systolic BP (SBP) and/or diastolic BP (DBP) values above the 95th percentile (z-score >1.65) adjusted for age, gender, and height [11]. Renal injury was defined as a reduced eGFR (<60 ml*min^−1^*1.73 m^−2^), proteinuria and/or high BP, and/or drug use to treat either proteinuria and/or high BP [12].

### KIMONO cohort

The KIMONO cohort comprises the worlds’ largest cohort of patients with an SFK. From the entire cohort of over 400 patients, the patients with matching characteristics were selected (as far as possible). Criteria for the selection consisted of the type of diagnosis (acquired SFK mainly based on the nephrectomy of a previously functional kidney) and duration of follow-up >10 years.

### Statistical analysis

All data were analyzed using SPSS Statistics 18.0 (SPSS, Chicago, IL, USA). Data were presented as number (%) or mean (standard deviation, SD). The BP z-scores were calculated using percentile scores [11]. Differences between measured z-scores and expected (z-score = 0) were calculated using the one sample *t*-test. Comparison in eGFR between different groups (gender, chemotherapy, disease stage) was performed using one-way ANOVA. Comparison between our cohort of WT survivors and the KIMONO cohort was carried out using the independent samples *t* test. Statistical significance was set at *p* < 0.05.

## Results

### Patient characteristics

**Fig. 1:**
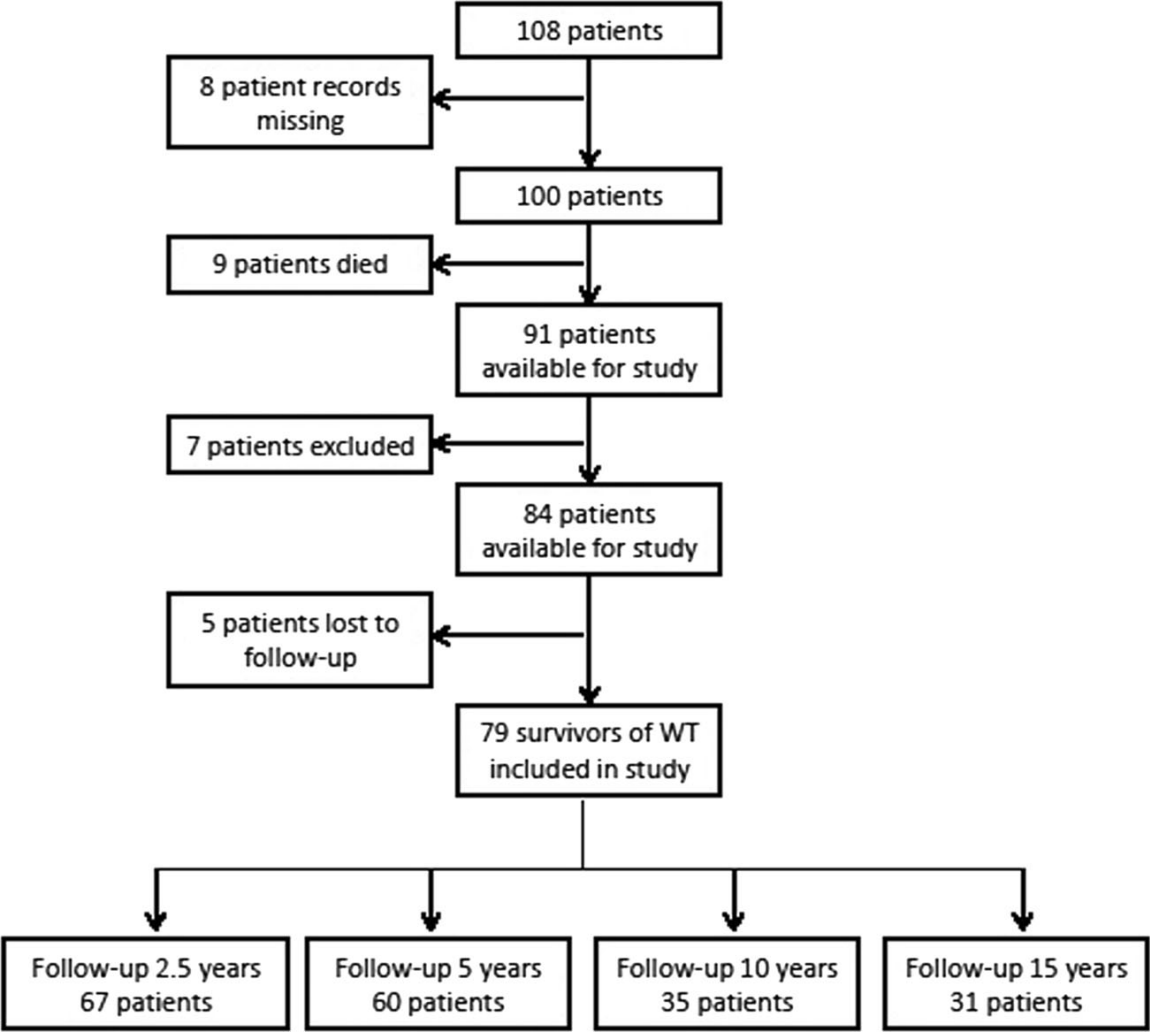
Patient inclusion criteria

We were able to identify 108 patients who have been treated for renal tumors at our institution. Figure 1 shows the inclusion criteria. Seven patients were excluded because of non-Wilms renal tumor (*n* = 3, clear cell sarcoma), bilateral WT (*n* = 3), and horseshoe kidney (*n* = 1). We were able to include 79 WT survivors in our study for whom follow-up details were available. Characteristics of the WT survivors and matched KIMONO subjects are shown in Table 1.

**Table 1:** Characteristics of Wilms tumor survivors and subjects from the Kidney of MONo-functional Origin (KIMONO) cohort

Patient characteristics	Wilms tumor	KIMONO	*p*
Number	79	184	
Gender			0.04
Male	40 (50 %)	118 (64 %)	
Female	39 (50 %)	66 (36 %)	
Age at nephrectomy (years)	3.7 (SD 2.5)	4.2 (SD 4.6)	0.4
Age at latest follow-up (years)	12.4 (SD 5.9)	10.5 (SD 6.0)	0.02
Follow-up duration (years)	9.1 (SD 5.7)	6.9 (SD 5.1)	0.003
Side of tumor			
Left	44 (56 %)		
Right	35 (44 %)		
Wilms tumor stage			
I	39 (49 %)		
II	15 (19 %)		
III	17 (22 %)		
IV	8 (10 %)		
Histology at diagnosis			
Low risk	9 (11 %)		
Intermediate risk	54 (69 %)		
High risk	8 (10 %)		
Treatment protocol^a^			
SIOP 9	13 (17 %)		
SIOP 93-01	31 (39 %)		
SIOP 2001	35 (44 %)		
Radiation therapy^b^	19 (24 %)		
Reason for poor kidney function leading to nephrectomy			
Congenital anomalies		32 (18 %)	
Obstructive uropathy		41 (22 %)	
Urinary tract infection		77 (42 %)	
Posterior urethral valves		14 (7 %)	
Miscellaneous		20 (11 %)	

Data are presented as mean (standard deviation, SD) or as a number (%)

*SIOP* International Society of Pediatric Oncology

^a^None of the patients was treated with ifosfamide

^b^None of the patients received radiation therapy to the remaining kidney

### Renal function of WT survivors

Chart review of our WT survivors showed that renal functional analysis was mainly performed using SBP, DBP, and eGFR. Unfortunately, there was insufficient data on proteinuria. Table 2 shows renal function parameters of WT survivors at the different follow-up time points. At diagnosis, mean SBP z-score was 2.1 (SD 1.4) and DBP z-score was 1.8 (SD 1.3). At follow-up, blood pressure returned to normal values. At discharge from the hospital, mean SBP and DBP z-scores were 0.3 (SD 1.3) and 0.2 (SD 0.7) respectively. During follow-up, BP z-scores remained stable on average and none of the survivors used renoprotective drugs during follow-up. The eGFR remained stable over the follow-up period. There were no significant differences in eGFR within the different treatment protocols, gender or disease stage (data not shown), nor was there an effect of radiation therapy.

**Table 2:** Renal function of Wilms tumor survivors at the different follow-up time points

	Diagnosis	Discharge	2.5 years	5 years	10 years	15 years
Age (years)	3.6 (2.4)	3.7 (2.5)	6.3 (2.5)	8.9 (2.8)	14.1 (2.5)	17.8 (3.6)
Systolic blood pressure (z-score)	2.1 (1.4)	0.3 (1.3)	0.0 (0.9)	0.2 (1.0)	0.6 (1.5)	0.6 (1.5)
Diastolic blood pressure (z-score)	1.8 (1.3)	0.2 (0.7)	0.8 (1.0)	0.1 (0.6)	0.0 (0.7)	0.0 (0.7)
eGFR (ml*min^−1^*1.73 m^−2^)	90 (36)	86 (21)	86 (25)	89 (23)	82 (22)	83 (14)

Data are presented as mean (standard deviation, SD)

*eGFR* estimated glomerular filtration rate

### Comparison with KIMONO study data

We compared renal function of WT survivors with a matched group of SFK subjects from the KIMONO cohort (Table 3). Despite the matching, the KIMONO patients were younger than the WT survivors. The eGFR was similar in the cohort of WT survivors and the KIMONO cohort and no significant difference in SBP and DBP z-scores between the two cohorts was noted. However, progressive renal injury was more frequently found in the KIMONO cohort (54 %) than in the WT survivors (23 %) after a follow-up of over 10 years.

**Table 3:** Renal function of Wilms tumor survivors at a follow-up of 15 years compared with the KIMONO cohort

	Wilms tumor	KIMONO	*p*
Number	31	57	
Age	17.8 (SD 3.6)	15.4 (SD 2.9)	0.002
Systolic blood pressure (z-score)	0.7 (SD 1.5)	0.3 (SD 1.1)	0.3
Diastolic blood pressure (z-score)	0.0 (SD 0.7)	0.0 (SD 0.8)	0.9
High blood pressure	6/23 (26 %)	6/57 (11 %)	0.08
eGFR (ml*min^−1^*1.73 m^−2^)	83 (SD 14)	89 (SD 31)	0.2
eGFR <60 ml*min^−1^*1.73 m^−2^	1/24 (4 %)	7/57 (12 %)	0.3
Proteinuria	ND	25/57 (44 %)	
Anti-hypertensive and/or anti-proteinuric drug use	0/31 (0 %)	20/57 (35 %)	<0.001
Renal injury	7/31 (23 %)	31/57 (54 %)	0.004

Renal injury was defined as high blood pressure, proteinuria, and/or the use of drugs treating either condition, and/or eGFR < 60 ml*min^−1^*1.73 m^−2^

Data are presented as mean (standard deviation, SD) or as number (%)

*eGFR* estimated glomerular filtration rate,* ND* not determined

## Discussion

In this study, we showed that progressive renal injury in WT survivors with SFK occurs in a similar manner to that in children with SFK of non-oncological causes, even though the frequency may be lower. Recently, a large cohort of more than 400 children with SFK was evaluated for signs of progressive renal injury (KIMONO) [2]. We were able to compare a smaller cohort of WT survivors with a matched group from this KIMONO cohort. This comparison showed that there was a trend toward a higher frequency of high BP in the WT survivors. However, none of the WT survivors was treated with anti-hypertensive/anti-proteinuric drugs compared with 35 % of the KIMONO cohort, which will have influenced the BP readings at follow-up. In addition, proteinuria and/or albuminuria was not tested in most WT survivors, whereas 44 % of individuals in the KIMONO cohort were found to have proteinuria. These missing data may account for a large proportion of the difference in frequency of progressive renal injury between the two cohorts. Another explanation for the identified difference in renal injury may be the high prevalence of associated congenital anomalies of the kidney and urinary tract in patients from the KIMONO cohort. Indeed, it has been shown that these associated malformations are an independent risk factor for progressive renal injury [2]. Finally, a selection bias in the KIMONO cohort may have accounted for a larger than expected number of individuals with progressive renal injury. The KIMONO cohort may be considered a biased cohort, as not all patients were included after a prenatal diagnosis, but may have been diagnosed based on clinical characteristics (e.g., recurrent urinary tract infections or a palpable abdominal mass). Even though previous analyses of such bias have not been shown to result in different conclusions, this has to be taken into account.

No difference in eGFR was found between the WT survivors and the KIMONO cohort. As most WT patients received drugs that were not directly nephrotoxic and the number of high-risk patients who received carboplatin was relatively small, this was in line with our expectations. The eGFR was not significantly different between the different treatment groups. However, subgroups of children treated with different chemotherapeutic drugs were small; therefore, we may have missed differences based on the sample size.

Green et al. recently reviewed the available evidence on progressive renal injury after successful treatment for ipsilateral nonsyndromic WT [6]. The risk of end-stage renal disease (ESRD) 20 years after diagnosis among children with unilateral, nonsyndromic WT treated according to the National Wilms Tumor Study Group protocols was 0.7 % [13]. Progressive renal injury in WT survivors of a less serious degree is reported to be up to 30 % in survivors without abdominal irradiation and up to 40 % in WT survivors with abdominal irradiation. In a large cohort of adult survivors of different types of childhood cancer, low eGFR was associated with treatment with unilateral nephrectomy, abdominal radiation therapy, cisplatin, and ifosfamide [14]. Based on data from reports from recent years (Table 4), hypertension and albuminuria are found in 13 % of WT survivors at latest follow-up, and 3 % had a clinically relevant reduced GFR. As most reports do not describe a concurrence of renal injury markers, it may be estimated from the recent literature that progressive renal injury is found in ±25 % of WT survivors, which is in line with our results.

**Table 4:** Frequency of progressive renal injury at the latest follow-up in Wilms tumor survivors based on published cohorts since 2005

	Number	Follow up duration (years)	Hypertension	Albuminuria or proteinuria	Medication use	eGFR < 60 ml*min^−1^*1.73 m^−2^	Renal injury* overall
Cozzi et al. 2005 [28]	16	6.0 (SD 3.4)	2/16 (12.5 %)	2/16 (12.5 %)	NR	NR	NR
Daw et al. 2009 [29]	11	11.2	2/11 (18.2 %)	2/11 (18.2 %)	NR	NR	NR
Stefanowicz et al. 2011 [30]	32	9.3 (SD 5.4)	2/32 (6.3 %)	7/32 (21.9 %)	0/32	0/32	NR
Sanpakit et al. 2013 [31]	17	4.8	NR	NR	NR	2/17 (11.8 %)	NR
Elli et al. 2013 [32]	25	9.9	3/15 (20.0 %)	NR	0/25	0/25	NR
Kern et al. 2014 [33]	55	6.3	NR	NR	NR	2/55 (3.6 %)	NR
Spreafico et al. 2014 [34]	15	13.3	0/15	2/15	NR	0/15	NR
Kishore et al. 2015 [35]	29	4.8 (SD 2.6)	2/29 (6.9 %)	1/29 (3.4 %)	NR	1/29 (3.4 %)	NR
Current report	31	14.8 (SD 3.3)	6/23 (26.1 %)	NR	0/31	1/24 (4.2 %)	7/31 (22.6 %)
Overall	231		17/141 (12.1 %)	14/103 (13.6 %)	0/88	6/197 (3.0 %)	

*eGFR* estimated glomerular filtration rate, *NR* not reported

^a^Renal injury was defined as a reduced eGFR (<60 ml*min^−1^*1.73 m^−2^), proteinuria/albuminuria, and/or hypertension, and/or drug use to treat either proteinuria and/or hypertension

In daily pediatric care, equations that use creatinine to obtain an eGFR have been widely adopted by clinicians, as recommended by the Kidney Disease Outcome Quality Initiative (K/DOQI) guidelines [12]; however, it has several limitations compared with the gold standard measurement of GFR by inulin clearance [15]. Westland et al. examined the precision of six common estimating equations in predicting the gold standard GFR, determined by an inulin single-injection method, in children with an SFK [16]. They concluded that the combined serum cystatin C/serum creatinine/blood urea nitrogen (BUN) Chronic Kidney Disease in Children (cKiD) equation estimates the GFR of children with an SFK with superior precision. However, when cystatin C is not available, eGFR–Schwartz is an acceptable alternative. In most studies reporting on progressive renal injury in WT survivors, GFR has been estimated using the Schwartz equation, as in our study. Different cut-off values of eGFR are used to define renal injury. Green et al. [6] used an eGFR of < 80 ml*min^−1^*1.73 m^−2^ to define renal injury, whereas we used an eGFR of < 60 ml*min^−1^*1.73 m^−2^, as was recommended by the clinical practice guidelines for chronic kidney disease in children and adolescents [12]. However, it must be noted that the decline in eGFR due to glomerular hyperfiltration is a relatively late phenomenon [17]. To detect early deterioration in eGFR, it would therefore be interesting to investigate more sensitive renal markers (such as fibroblast growth factor [FGF] 23) in WT survivors during follow-up.

With excellent survival rates of WT patients reaching 90 %, the identification and prevention of late effects due to treatment are increasingly important. Life-long monitoring of WT survivors based on the knowledge of the present study and of previously described studies is warranted.

Nephron-sparing surgery (NSS) in unilateral WT patients could potentially diminish progressive renal injury because of the loss of nephrons [18]. Upfront chemotherapy and NSS have been shown to provide safe and effective oncological control, while optimizing renal function in patients with bilateral WT [19, 20]. Hubertus et al. showed that hypertension is less common in patients with bilateral WT after bilateral NSS. At the same time, rates of local relapse or disease-associated death were equal to those of patients with bilateral WT and unilateral NSS and contralateral nephrectomy, suggesting NSS to be oncologically safe [21]. A recent Surveillance, Epidemiology, and End Results (SEER) analysis on the use of NSS and the impact on survival in children with WT also concluded that overall survival was similar between NSS and radical nephrectomy in a cohort of both bilateral and unilateral WT patients [22]. Cost et al. compared renal function outcomes after NSS and radical nephrectomy in nonsyndromic unilateral WT patients only. EGFR at last follow-up was equal in the two groups [23]. Data from the Internatioanal Society of Pediatric Oncology (SIOP) 2001 study showed that NSS was only performed in 3 % of patients with unilateral WT (65 % in stage I tumors). The authors concluded that despite excellent survival with few relapses, the gain of nephrons needs to be weighed against the risk of inducing stage III with intensified therapy [24]. Studies on renal function after NSS are based on relatively small patient groups and are all of a retrospective nature. Larger prospective studies are needed to fully address the gain of renal function versus oncological outcome in NSS.

Preserving renal function may serve another important goal, namely preventing cardiovascular complications. Cardiorenal syndrome is defined as a pathological disorder of the heart and kidneys in which acute or chronic dysfunction in one organ may induce acute or chronic dysfunction in the other organ. It includes a broad spectrum of diseases in which the heart and kidney are both involved [25]. Patients with chronic kidney disease are at a higher risk of mortality than the general population. The renal dysfunction represents an independent risk factor for cardiovascular disease, as these patients present higher mortality rates for myocardial infarction and sudden death [26, 27].

Our study has several limitations. First of all, our study has a retrospective design. As a pre-defined follow-up schedule has only been implemented in the last few years, data were missing from a different number of patients at each time-point. In addition, some data were retrieved from other hospitals, where other methods and reference values may have been used. BP was measured manually at the beginning of the study period, and by using oscillometric methods (DynaMap) for the last few years. Unfortunately, information on the method of measurement is not mentioned in the patient record, which may have an effect on the results. In addition, BP used to be measured only once, whereas it is measured three times in the current follow-up protocol. Analogous to the guideline that was presented for children with an SFK [17], we suggest a similar follow-up protocol for WT survivors with an SFK (Table 5).

**Table 5:** Opinion-based recommendation for the clinical follow-up of Wilms tumor survivors with a solitary functioning kidney

	No progressive renal injury	eGFR < 60 ml*min^−1^*1.73 m^−2^ and/or medication for proteinuria/hypertension
Blood pressure	Once yearly	2–4/year
Albuminuria	Once yearly	2–4/year
Serum creatinine/GFR	Every 5 years	2–4/year

24-h ambulatory blood pressure measurement is preferred in children and adults

Albuminuria may be estimated through the urinary albumin-to-creatinine ratio and should be determined in a first fresh morning sample (normal value <30 mg/g)

GFR can be estimated using the commonly used Schwartz formula (see Materials and methods)

Last ultrasound to be performed at 15–16 years of age

*eGFR* estimated glomerular filtration rate

## Conclusion

In a similar fashion to children with an SFK for non-oncological reasons, progressive renal injury presents in WT survivors, but the incidence is lower. However, renal injury in our cohorts may have been underestimated owing to the absence of information on proteinuria in WT survivors. As with patients with a non-malignant SFK, long-term follow-up is essential to monitor, inform, and prevent disease sequelae for WT survivors. A standardized follow-up protocol (including urinalysis) has been put in place to optimize follow-up.
